# Early Detection of Dementia Through Spectralis Optical Coherence Tomography in a Taiwanese Cohort

**DOI:** 10.3390/diagnostics16040534

**Published:** 2026-02-11

**Authors:** Man Sze Wong, Yung-Chuan Huang, Chao-Wei Wu, Yue-Cune Chang, Hsin-Yi Chen

**Affiliations:** 1 Department of Medical Education, National Taiwan University Hospital, Taipei 100, Taiwan; mancycat1@ntuh.gov.tw; 2 School of Medicine, College of Medicine, Fu Jen Catholic University, New Taipei City 242062, Taiwan; richardych1111@gmail.com; 3 Department of Neurology, Fu Jen Catholic University Hospital, New Taipei City 242062, Taiwan; 4 Cheng Ching Eye Center, Kaohsiung City 807, Taiwan; chaowei196@gmail.com; 5 Department of Mathematics, Tamkang University, New Taipei City 25137, Taiwan; ychang@math.tku.edu.tw; 6 Department of Ophthalmology, Fu Jen Catholic University Hospital, New Taipei City 24352, Taiwan; 7 School of Medicine, College of Medicine, Kaohsiung Medical University, Kaohsiung City 80756, Taiwan

**Keywords:** Spectralis optical coherence tomography, cognitive disorders, diagnosis

## Abstract

**Background and Objectives:** Dementia is an essential neurodegenerative disease with pathologic changes in the central nervous system, but also the retina. To evaluate the diagnostic performance of Spectralis optical coherence tomography (OCT) parameters for mild cognitive impairment (MCI) and mild dementia in an Asian population from Taiwan. **Methods**: This retrospective cross-sectional study evaluated 43 patients with MCI (mean deviation [MD]: −5.05 ± 4.25 dB), 13 patients with mild dementia (MD: −9.03 ± 6.66 dB), and 32 healthy controls (MD: −2.50 ± 2.12 dB). OCT was performed on both eyes of each subject. The diagnostic sensitivity in identifying individuals with cognitive impairment of the Spectralis OCT parameters—such as those of the optic nerve head and macula—was compared across these groups. The area under the receiver operating characteristic curve (AUC) for each parameter was calculated to assess its sensitivity in differentiating between healthy eyes and those of individuals with MCI or mild dementia. **Results**: Among the parameters evaluated, the Bruch’s membrane opening minimum rim width (BMO-MRW) nasal inferior region (ACU = 0.720) was the optimal parameter for distinguishing individuals with MCI from healthy controls. However, the highest AUC of 0.861 was achieved through a combination of five parameters. In distinguishing individuals with mild dementia from healthy controls, the BMO-MRW temporal superior region (ACU = 0.764) was the optimal parameter, with an AUC of 0.940 after adjusting for age and MD. Moreover, the condition of the macular nerve fiber layer outer inferior parameter moderately predicted disease progression (AUC = 0.713). **Conclusions:** Our preliminary data demonstrate that Spectralis OCT shows potential in detecting MCI and mild dementia as well as for assessing disease progression in a Taiwanese population. Additional large-scale longitudinal and multiracial studies are essential to validate these findings.

## 1. Introduction

Although neurocognitive disorders comprise a spectrum of conditions such as delirium, mild cognitive impairment (MCI), and dementia, Alzheimer’s disease (AD) remains the leading cause of dementia in older populations. Several clinical assessments are employed to detect and monitor the progression of AD, such as the Mini-Mental State Examination (MMSE) [1], phosphorylated tau181 (P-tau181) in plasma [2], and amyloid-β (Aβ) counts in positron emission tomography imaging or cerebrospinal fluid analysis [3]. Although these biomarkers were integrated into a diagnostic tool for AD, they are not widely available or used in routine practice worldwide. When exploring the biological processes of AD, the neuropathologic burden may precede the onset of the disease by 10 to 20 years. Current research targets improving early detection and treatment of MCI, particularly in patients at high risk for progression to dementia [4]. Consequently, early clinical diagnosis and intervention are crucial to preserving cognitive function and delaying disease progression in individuals with AD [5,6]. Early-stage AD, often referred as mild cognitive impairment (MCI) [7], is increasingly recognized as a critical therapeutic target, although identifying MCI in a timely manner is challenging [4,6].

The Diagnostic and Statistical Manual of Mental Disorders, Fifth Edition (DSM-V) characterizes neurocognitive disorders across six domains, one of which is perceptual motor defect [8]. Multiple visual problems have been documented in patients with AD [9]. For example, Hinton et al. reported substantial degeneration of retinal ganglion cells in the optic nerves of patients with AD compared with age-matched healthy controls [10]. Technologies such as spectral domain optical coherence tomography (SD-OCT) are noninvasive and enable assessments of retinal fiber nerve layers. Additionally, one study suggested that OCT parameters can be used to identify both AD and MCI [11]. However, studies reporting the ability of the Spectralis OCT device to detect MCI and mild dementia in the early stages are lacking, particularly studies assessing these conditions in Asian populations. The present study used a Spectralis OCT to identify dementia in a Taiwanese population.

## 2. Materials and Methods

### 2.1. Study Design

This study enrolled patients with symptoms of cognitive impairment evaluated over the period from July 2022 to July 2023. This study was approved by the Institutional Review Board of Fu Jen Catholic University Hospital (FJUH) (approval numbers FJUH111198, Date 7 July 2021) and was conducted in accordance with the ethical principles stipulated in the Declaration of Helsinki. FJCUH is a teaching hospital with a dementia care service center located in New Taipei City, Taiwan. Patients with degenerative dementia may be referred to FJCUH for clinical evaluation and management. The diagnosis of dementia was based on the definition of the DSM-V and was determined by at least one neurologist and one psychologist. The stages of dementia were classified according to the Clinical Dementia Rating (CDR) score at baseline. The patients with CDR scores of 0.5 were assessed as having MCI, whereas those wit h CDR scores of 1 were assessed as having mild dementia. The MCI group consists of participants with mild memory dysfunction and preserved daily activity ability. Patients were excluded if they had structural lesions in brain imaging that could result in cognitive decline or if they had had abnormal laboratory results, such as apparent renal and liver function abnormalities, electrolyte imbalance, infection, vitamin B12 deficiency, or hypothyroidism causing consciousness disturbance. Patients with a history of mental or psychiatric disorders that could mimic dementia were also excluded.

The patients were regularly followed up at FJCUH and received cognitive re-evaluations in the year after diagnosis. These cognitive evaluations employed the Taiwanese Mental State Examination—a version of the Mini-Mental State Examination (MMSE)—and CDR scores [12]. Dementia progression was defined as an increase in CDR scores on the re-evaluation.

### 2.2. Spectralis OCT Imaging

Radial and circular scans and posterior pole horizontal scans were conducted on the optic nerve head (ONH) and macula by using the Spectralis OCT device (Heidelberg Engineering, Heidelberg, Germany) [13]. Information on the ONH, specifically the Bruch’s membrane opening minimum rim width (BMO-MRW) and the thickness of the circumpapillary retinal nerve fiber layer (cpRNFL), was obtained through these radial and circular scans. The shortest distance between the Bruch’s membrane opening (BMO) and the internal limiting membrane under transverse analysis with radial B-scans was defined as the BMO-MRW. To determine the cpRNFL thickness, three circular retinal nerve fiber layer (RNFL) scans were conducted at diameters of 3.5, 4.1, and 4.7 mm. Only data from the 3.5 mm diameter scans were analyzed. The results are presented in micrometers as the global average of the measurements and six Garway–Heath sectors. The six Garway–Heath sectors were defined by their angular degrees around the eye as follows: 315° to 45° (temporal), 45° to 85° (temporal superior), 85° to 125° (nasal superior), 125° to 235° (nasal), 235° to 275° (nasal inferior), and 275° to 315° (temporal inferior).

A posterior pole protocol was employed to generate a 30° × 25° OCT volume scan on an 8 × 8 grid over the fovea–disk axis by using the early treatment diabetic retinopathy study (ETDRS) grid mode. The 64 sections of the grid spanned the top and bottom and the temporal and nasal sides. The ETDRS scans yielded similar results to those of the circular RNFL scans, revealing three rings with diameters of 1, 3, and 6 mm, respectively. These rings were subsequently segmented into nine sections on the basis of their diameters. The central thickness was defined as the thickness of the 1 mm inner ring. The 3 mm intermediate and 6 mm outer rings were further divided into four quadrants. The quadrants from the intermediate ring were labeled inner temporal (T1), inner inferior (I1), inner nasal (N1), and inner superior (S1). Similarly, the 6 mm outer ring was divided into outer temporal (T2), outer inferior (I2), outer nasal (N2), and outer superior (S2) quadrants. The retinal layers in the quadrants were automatically segmented into 10 components. Because segmentation errors are common in cpRNFL and macular scans [14], the raw B-scan images were screened for artifacts, and those with substantial artifacts were excluded. The exclusions involved 8.8% of the eyes of those with MCI and 16.2% of the eyes of those with mild dementia.

### 2.3. Statistical Analysis

The baseline characteristics of the healthy control, MCI, and mild dementia groups were compared using Pearson’s chi-square test, and an independent *t* test was used to compare individuals with MCI and those with mild dementia. The average result of both eyes was recorded. We included only one eye from each patient in the comparison. To determine the diagnostic sensitivity of each parameter in identifying cognitive impairment across groups, we calculated the areas under the receiver operating characteristic curves (AUCs), and 95% confidence intervals. We maximized the discriminatory ability of the model through a multiple logistic regression with generalized estimating equations; an exchangeable working correlation matrix was used as the parameter-based classifier. SPSS (v.26; IBM Corp, Armonk, NY, USA) was used to conduct the analyses, and significance was set at *p* < 0.05.

## 3. Results

### Demographic and Clinical Data

Table 1 presents the baseline characteristics of the 88 participants. A total of 32 eyes from healthy individuals (mean deviation [MD]: −2.50 ± 2.12 dB), 43 eyes from patients with MCI (mean deviation [MD]: −5.05 ± 4.25 dB), and 13 eyes from patients with mild dementia (mean deviation [MD]: −9.03 ± 6.66 dB) were included. The healthy control group was significantly younger than the other groups. Statistically significant differences were also observed in sex and hypertension. By contrast, no significant differences were observed in intraocular pressure, refraction error, or diabetes mellitus among the groups.

Table 2 compares patient retinal thickness using the optimal parameters to distinguish among individuals with MCI, those with mild dementia, and those without dementia. In differentiating the MCI group from the controls, the nasal inferior (NI) region of the BMO-MRW exhibited the optimal sensitivity (AUC = 0.720). Parameters in the macular area using the ETDRS mode or posterior pole asymmetry analysis (PPAA) exhibited fair sensitivity (AUC < 0.7). The next most sensitive parameter was the retinal average thickness (RAT, 1.6), which had an AUC of 0.653 in the 8 × 8 grid mode under PPAA (Table S1). The temporal superior BMO-MRW most clearly distinguished individuals with mild dementia from healthy controls, exhibiting an AUC of 0.764. Macular parameters under the ETDRS mode also exhibited fair sensitivity (AUC < 0.7). In the 8 × 8 grid mode under PPAA, the optimal distinguishing parameter was RAT 4.1 (AUC = 0.708; Table S2). In differentiating MCI from mild dementia, the optimal parameter was the inner nasal (N1) macular ganglion cell layer (GCL) (AUC = 0.723; Table S3).

**Figure 1:**
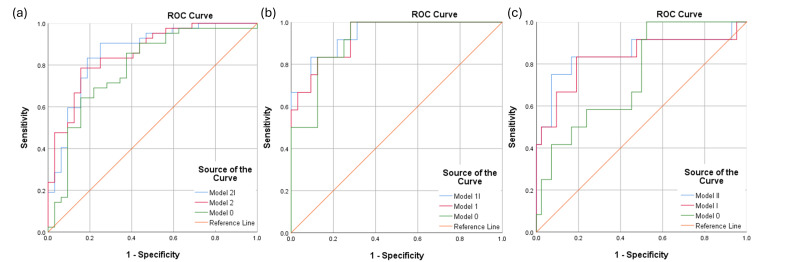
Receiver operating characteristic curves comparing the optimal combinations of parameters for screening individuals (**a**) with MCI, (**b**) mild dementia, and (**c**) MCI versus mild dementia.

To maximize the model’s discriminatory ability, the parameters were adjusted for confounding variables by using a generalized estimating equation multiple logistic regression exchangeable working correlation matrix (Table 3 and Figure 1). In distinguishing individuals with MCI from healthy controls, the combination of age, pattern standard deviation, mean deviation, BMO-MRW_NI, and RAT 1.6 (Model 2I) had an AUC of 0.861. The combination of age, mean deviation, and BMO-MRW_TS (Model 1I) most accurately distinguished individuals with mild dementia from healthy controls (AUC = 0.940). In differentiating individuals with MCI from those with mild dementia, the combination of age, refraction, mean deviation, hypertension, GCL_N1, and whole retina_T2 (Model II) yielded an AUC of 0.851.

**Figure 2:**
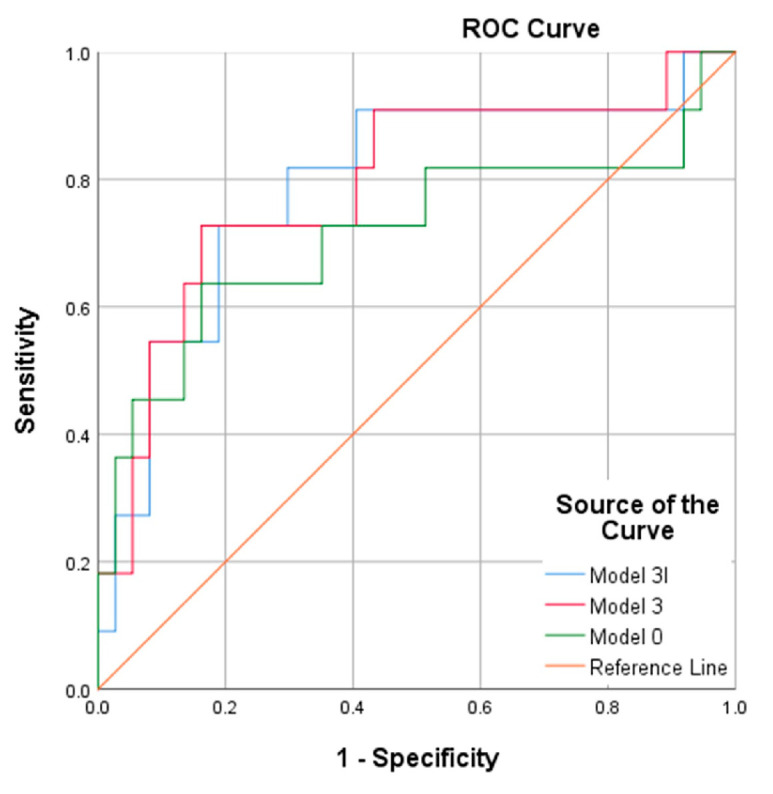
Receiver operating characteristic curves comparing the optimal combinations of parameters for screening patients with and without disease progression.

The OCT parameters were also evaluated for their ability to predict disease progression. The baseline characteristics of the patients are listed in Table 4 and Table S4. Data on five patients who were lost to follow-up were excluded. The 51 remaining patients were subdivided into two groups (12 with disease progression and 39 without disease progression). The individuals who experienced disease progression had higher rates of diabetes mellitus and higher CDR scores than those whose condition did not progress. The results presented in Table 5 reveal that the macular parameter NFL_I2 had an AUC of 0.713 in detecting dementia progression. However, when patient age, ocular refraction, mean deviation, hypertension, and diabetes mellitus were considered (Model 3I; Table 6 and Figure 2), the AUC increased to 0.791.

## 4. Discussion

Neuronal damage is common in individuals with neurocognitive disorders [15]. Nevertheless, such damage is not confined to the central nervous system but also affects the retinal axons [16]. Therefore, OCT can be used to capture cross-sectional images of ocular structures, revealing retinal thickness and damage to the retinal vascular network.

Retinal thickness is a biomarker of neurocognitive disorders such as MCI [17,18,19,20,21], AD [11,22,23,24,25,26,27], Huntington’s disease [28], Parkinson’s disease [29,30], frontotemporal dementia [31] and cerebral autosomal dominant arteriopathy with subcortical infarcts and leukoencephalopathy [32,33]. Studies have also reported that retinal blood flow, especially in the macular region, is correlated with the loss of the ganglion cell inner plexiform layer (GCIPL) [18,19,20], suggesting that a neurovascular–hemodynamic mechanism underlies the pathogenesis and progression of dementia [34,35]. Additionally, Armstrong proposed that functional and morphological alterations in the retina may occur several years prior to the clinical onset of AD and suggested that OCT can detect these early biomarkers of cognitive decline [36].

In individuals with dementia, the thickness of the peripapillary RNFL (ppRNFL) and the macula—which contains more than 50% of retinal ganglion cells (RGCs)—has been extensively studied. For example, Ito et al. suggested that macular thickness may more clearly indicate neurodegenerative damage than the ppRNFL [37,38]. Notably, the present study revealed no significant changes in the ppRNFL thickness of the patients with MCI compared with that of healthy controls. However, Kesler reported a significant reduction in RNFL thickness in individuals with AD and MCI compared with controls, particularly in the inferior quadrants of the ONH, although the superior quadrants were significantly thinner only in the individuals with AD [39]. Additionally, the study of Shen et al. indicated that the thickness of the inferior quadrant ppRNFL is associated with episodic memory function in patients with MCI [40]. Therefore, the inferior quadrant of the RNFL may be an indicator of the early stages of neurocognitive disorders. However, this interpretation warrants caution because the change in ppRNFL thickness reported in individuals with MCI and AD is inconsistent across studies [41].

Among available SD-OCT units, the Spectralis device alone evaluates the BMO-MRW, elucidating the mechanisms of retinal axonal damage by accurately defining the anatomical border of RGC axons. This imaging modality has been reported to enhance diagnostic accuracy in evaluating individuals with glaucoma [13,42]. Similarly, the present study revealed that the NI BMO-MRW (AUC = 0.720) and temporal superior BMO-MRW (AUC = 0.764) were the optimal parameters for distinguishing individuals with MCI and mild dementia from healthy controls. This study demonstrates the potential of the BMO-MRW to detect MCI and mild dementia early. However, additional large-scale longitudinal studies are required to verify these findings.

Several studies have noted the accuracy of OCT in distinguishing MCI from mild dementia. For example, one study conducted in Asia reported that the ppRNFL thickness of the superior quadrant gradually decreased as individuals progressed from MCI to severe AD [43]. Another study conducted in Asia reported decreased thickness of the RNFL and reduced macular volume in patients with AD and MCI. However, no correlation between these changes and the severity of dementia was uncovered [21]. Similarly, the present study revealed a lack of significant differences in all RNFL region thicknesses but the RNFL-TI area (Table S3), a result consistent with those of studies evaluating populations of European descent [44,45]. Due to the inconsistency of the evidence, the association between RNFL thickness and the severity of dementia cannot be readily determined. However, one study conducted in South Korea [46] reported that macular GCIPL thickness is a promising biomarker of the progression of MCI and AD, a result comparable to the finding of the present study that macular GCL-N1 (AUC = 0.723) optimally discriminates between individuals with MCI and those with mild dementia.

Studies have suggested that patients with AD have a thinner ppRNFL than those with MCI or mild dementia do; this thinness is especially pronounced in the temporal superior [47] and superior regions [22,39,43,48]. By contrast, the present study revealed thinner regions in the BMO-MRW than in the ppRNFL. This discrepancy may be attributable to the modest average difference (4–12%) reported in the ppRNFL thickness between the individuals with AD and the controls, suggesting that macular measurements may be more reliable indicators of visual impairment than retinal measurements [41,49]. Additionally, the Spectralis OCT device used in the present study enabled us to conduct PPAA, which evaluates 64 parameters [50]. PPAA uses an 8 × 8 grid to enhance the ability of the instrument to assess dementia severity. Notably, RAT 4.1 exhibited the greatest discriminatory ability after the macula (AUC = 0.708). These results suggest that the BMO-MRW serves as a biomarker of early dementia.

To enhance the predictive accuracy of our models, we conducted a multivariate regression that integrated OCT parameters, ocular measurements, and various comorbidities. The optimal combinations of parameters for distinguishing between individuals with MCI and controls, those with early dementia and controls, and those with MCI and early dementia had AUCs of 0.861, 0.940, and 0.851 respectively. Similarly, Larrosa et al. reported an AUC of 0.967 in distinguishing between White individuals with AD and healthy controls [51]. Furthermore, the study of Chua J et al. [52]. reported that compensated RNFL outperformed measured RNFL in discriminating between individuals with MCI and those with AD (AUC = 0.74 vs. 0.69; *p* = 0.026). They also reported that combining macular and compensated cpRNFL parameters yielded the highest AUC in distinguishing between individuals with AD and those with MCI (AUC = 0.80 vs. 0.69; *p* < 0.001).

The present study assessed changes in cognitive function in all the patients with MCI and dementia at a 1-year follow-up to elucidate the role of retinal OCT measurements in predicting disease progression. An earlier study reported that a thinner RNFL was associated with an increased risk of dementia in a Dutch adults [53]. Another study also demonstrated that ganglion cell and RNFL thicknesses were inversely correlated with AD duration and severity [25]. Similarly, in the present study, NFL_I2 optimally distinguished between individuals whose disease had progressed and those whose disease had not. Consistent with the findings of Kivipelto and Leibson [54,55], we observed that the combination of ocular measurements and various comorbidities had greater discriminatory ability compared with NFL_I2 alone (AUC of 0.791 vs. 0.713). Nevertheless, these findings are preliminary and warrant further investigation.

This study has several limitations. First, because this study was conducted in a real-world clinical setting, images with poor segmentation were manually excluded during the initial screening. Marquié et al. suggested that OCT image quality is reduced in individuals with cognitive decline [56], which may have introduced bias through the unintentional exclusion of individuals with severe cognitive impairment. Second, numerous variables such as age, sex, myopia, and comorbidities may have confounded the results [57,58,59,60]. Third, our study cohort is small with limited number of participants of Taiwanese population, which might limit the statistical power of the analysis. Therefore, our findings must be interpreted with caution.

Our study also has several strengths. First, we explored dementia by using Spectralis OCT measurements in an Asian population. This OCT device facilitated evaluations of the BMO-MRW of optic disk sections [61,62,63], enhanced resolution in macular segmentation [64,65], and included the Nsite Axonal Analytics module, which facilitated assessments of neurodegeneration [27]. Our findings can serve as a valuable reference for the early detection of dementia, especially in Asian populations with AD.

## 5. Conclusions

Our preliminary data demonstrate that Spectralis OCT shows potential in detecting MCI and mild dementia as well as for assessing disease progression in a Taiwanese population. Additional large-scale longitudinal and multiracial studies are essential to validate these findings.

## Figures and Tables

**Table 1:** Baseline Features of the Healthy Controls and Study Groups. (One Randomly Selected Eye).

Features	Healthy Controls (*n* = 32)	MCI (*n* = 43)	Mild Dementia (*n* = 13)	
	Mean ± SD	Mean ± SD	Mean ± SD	*p*
Age (years)	71.00 ± 4.49	74.56 ± 4.94	76.92 ± 6.34	0.003
Sex (male: female)	13:19	27:16	3:10	0.021
Hypertension, No. (%)	34.4%	43.00%	72.92%	0.034
Diabetes mellitus, No. (%)	31.3%	43.00%	53.85%	0.355
MMSE (* AD-8)	(*)	18.85 ± 5.78	16.29 ± 5.06	0.112 ^+^
Refraction (D)	−0.10 ± 2.43	0.31 ± 1.64	−0.41 ± 1.18	0.405
IOP	12.21 ± 4.21	13.02 ± 3.53	14.12 ± 4.06	0.322
Axial length	23.59 ± 1.21	23.68 ± 1.14	23.45 ± 0.71	0.773
ACD	3.28 ± 0.88	3.28 ± 0.82	3.72 ± 1.07	0.258
WTW	11.59 ± 0.46	11.51 ± 0.75	11.53 ± 0.44	0.891
MD (dB)	−2.50 ± 2.12	−5.05 ± 4.25	−9.03 ± 6.66	<0.001
PSD (dB)	2.69 ± 1.49	4.19 ± 2.04	5.54 ± 2.36	<0.001

ACD: Anterior chamber depth. IOP: Intraocular pressure. MCI: Mild cognitive impairment. MD: mean deviation. MMSE: Mini-Mental State Examination. PSD: Pattern standard deviation. WTW: White-to-white. * AD-8 (Alzheimer Disease Dementia Screening Interview Eight Item Version) was used in initial screening, with a score of 0–1 indicating healthy controls. ^+^ *t* test to distinguish between MCI and mild dementia.

**Table 2:** Comparison of Retinal Thickness of Patients Using the Optimal Parameter to Distinguish Among Patients With MCI, Those With Mild Dementia, and Healthy Controls.

MCI vs. Health Controls							
Scan	Best Parameter	Thickness (µm) (Mean ± SD)		*p* *	AUC (95% CI)	Sensitivity at 95% Specificity (%)	Sensitivity at 80% Specificity (%)
RNFL	Nasal-superior (NS)	124.01 ± 20.19	116.95 ± 22.78	0.289	0.603 (0.469, 0.736)	9.4	18.8
BMO-MRW	Nasal-inferior (NI)	309.28 ± 58.39	354.19 ± 54.93	0.086	0.720 (0.604, 0.836)	2.3	7.0
ETDRS							
RETINA	Outer inferior (I2)	275.70 ± 16.32	279.19 ± 17.73	0.154	0.606 (0.427, 0.711)	2.3	14.0
NFL	Inner nasal (N1)	24.70 ± 13.42	20.21 ± 2.46	0.272	0.610 (0.483, 0.738)	0.0	3.1
GCL	Central (C)	17.38 ± 9.58	14.53 ± 5.46	0.050	0.585 (0.454, 0.716)	0.0	12.5
IPL	Outer inferior (I2)	26.09 ± 4.84	26.79 ± 3.97	0.214	0.584 (0.451, 0.716)	2.3	11.6
PPAA	RAT_16	0.274 ± 0.02	0.285 ± 0.02	0.014	0.653 (0.525, 0.781)	2.3	4.7
**Mild dementia vs. Health controls**							
RNFL	Temporal (T)	84.08 ± 39.78	75.03 ± 14.24	0.317	0.659 (0.467, 0.850)	0.0	7.7
BMO-MRW	Temporal-superior (TS)	246.85 ± 40.02	293.06 ± 47.27	0.002	0.764 (0.623, 0.905)	0.0	0.0
ETDRS							
RETINA	Outer inferior (I2)	274.50 ± 22.68	279.19 ± 17.73	0.230	0.667 (0.494, 0.840)	15.4	23.1
NFL	Outer nasal (N2)	45.69 ± 12.49	18.35 ± 2.81	0.455	0.597 (0.418, 0.777)	7.7	15.4
GCL	Inner nasal (N1)	44.65 ± 8.86	31.74 ± 4.31	0.360	0.633 (0.444, 0.832)	7.7	15.4
IPL	Outer inferior (I2)	25.73 ± 4.02	26.79 ± 3.97	0.191	0.626 (0.438, 0.815)	0.0	15.4
PPAA	RAT_41	0.244 ± 0.02	0.253 ± 0.02	0.223	0.708 (0.528, 0.888)	7.7	7.7
**Mild dementia vs. MCI**							
RNFL	Temporal-inferior (TI)	159.27 ± 23.96	142.97 ± 20.92	0.020	0.695 (0.531, 0.859)	2.3	4.7
BMO-MRW	Temporal (T)	172.08 ± 23.28	189.80 ± 31.98	0.019	0.659 (0.508, 0.810)	0.0	0.0
ETDRS							
RETINA	Outer temporal (T2)	268.54 ± 19.63	278.91 ± 19.77	0.056	0.707 (0.528, 0.885)	7.7	15.4
NFL	Inner nasal (N1)	22.35 ± 11.60	24.70 ± 13.42	0.272	0.662 (0.494, 0.830)	7.7	7.7
GCL	Inner nasal (N1)	44.65 ± 8.86	48.36 ± 5.41	0.087	0.723 (0.532, 0.914)	7.7	15.4
IPL	Inner nasal (N1)	39.31 ± 8.09	40.52 ± 3.51	0.304	0.697 (0.497, 0.897)	7.7	23.1
PPAA	RAT_52	0.273 ± 0.03	0.281 ± 0.03	0.191	0.693 (0.507, 0.880)	7.7	15.4

AUC: area under the receiver operating characteristic curve. BMO-MRW: Bruch’s membrane opening minimum rim width. CI: confidence interval. ETDRS: early treatment of diabetic retinopathy study. GCL: macular ganglion cell layer. IPL: macular inner plexiform layer. MCI: Mild cognitive impairment. NFL: macular retinal nerve fiber layer. PPAA: posterior pole asymmetry analysis. RAT: retinal average thickness. RETINA: whole retinal layer. RNFL: circumpapillary retinal nerve fiber layer. *: Significance was set at *p* < 0.05.

**Table 3:** Optimal Combination of Parameters for Distinguishing Between Individuals With MCI or Mild Dementia and Healthy Controls.

Subtypes	Parameters Included	AUC (95% CI)
MCI vs. Normal	Model 2I: Age, PSD, MD, MRW (Nasal inferior), PPAA (RAT_16)	0.861 (0.773, 0.949)
	Model 2: Age, PSD, MRW (Nasal inferior), PPAA (RAT_16)	0.856 (0.771, 0.941)
	Model 0: Age, Refraction, PSD, MD	0.785 (0.675, 0.895)
Mild dementia vs. Normal	Model 1I: Age, MD, MRW (Temporal superior)	0.940 (0.870, 1.000)
	Model 1: Age, MD, MRW (Temporal superior), PPAA (RAT_41)	0.932 (0.858, 1.000)
	Model 0: Age, Refraction, PSD, MD	0.914 (0.832, 0.997)
MCI vs. Mild dementia	Model II: Age, Refraction, MD, Hypertension, GCL (Inner nasal), RETINA (Outer temporal)	0.851 (0.696, 1.000)
	Model I: Age, MD, Hypertension, GCL (Inner nasal), RETINA (Outer temporal)	0.831 (0.671, 0.992)
	Model 0: Age, Refraction, PSD, MD	0.744 (0.597, 0.891)

AUC: area under the receiver operating characteristic curve. CI: confidence interval. GCL: macular ganglion cell layer. MCI: Mild cognitive impairment. MD: mean deviation. MRW: Bruch’s membrane opening minimum rim width. PPAA: posterior pole asymmetry analysis. PSD: pattern standard deviation. RAT: retinal average thickness.

**Table 4:** Baseline Characteristics of Patients With and Without Disease Progression.

Features	With Progression (*n* = 12)	Without Progression (*n* = 39)	
	Mean ± SD	Mean ± SD	*p*
Age (years)	74.42 ± 5.93	75.92 ± 4.84	0.217
Sex (male:female)	4:8	22:17	0.087
Hypertension, No. (%)	50.00%	58.97%	0.303
Diabetes mellitus, No. (%)	66.67%	28.21%	0.014
Education (years)	5.58 ± 3.68	6.79 ± 4.12	0.172
MMSE	13.38 ± 2.97	19.40 ± 5.50	0.001
CDR	1.33 ± 0.49	0.55 ± 0.15	<0.001
Disease progress, No. (%)	100%	0%	<0.001
Refraction (D)	0.02 ± 1.27	0.26 ± 1.52	0.298
IOP	13.79 ± 3.47	13.24 ± 3.84	0.321
Axial length	23.53 ± 1.58	23.62 ± 0.95	0.423
ACD	3.33 ± 0.88	3.45 ± 0.95	0.343
WTW	11.48 ± 0.37	11.49 ± 0.79	0.467
MD (dB)	−8.63 ± 6.88	−5.55 ± 4.38	0.084
PSD (dB)	5.49 ± 2.04	4.40 ± 2.19	0.066

ACD: Anterior chamber depth. CDR: Clinical Dementia Rating. IOP: Intraocular pressure. MD: mean deviation. MMSE: Mini-Mental State Examination. PSD: Pattern standard deviation. WTW: White-to-white.

**Table 5:** Comparison of Patient Retinal Thickness Using the Optimal Parameters to Distinguish Between Patients With and Without Disease Progression.

Scan	Best Parameter	Thickness (µm) (Mean ± SD)		*p* *	AUC (95% CI)	Sensitivity at 95% Specificity (%)	Sensitivity at 80% Specificity (%)
		With Progression	Without Progression				
RNFL	Temporal-superior (TS)	140.71 ± 14.98	133.24 ± 21.15	0.093	0.641 (0.476, 0.806)	10.3	20.5
BMO-MRW	Nasal (N)	272.13 ± 49.31	280.97 ± 51.74	0.299	0.573 (0.389, 0.756)	0.0	16.7
ETDRS							
RETINA	Inner inferior (I1)	323.50 ± 34.42	328.90 ± 23.90	0.310	0.670 (0.468, 0.871)	8.3	8.3
NFL	Outer inferior (I2)	26.04 ± 13.37	38.35 ± 6.42	0.447	0.713 (0.521, 0.904)	8.3	33.3
GCL	Outer inferior (I2)	33.04 ± 6.74	30.41 ± 5.51	0.118	0.653 (0.433, 0.872)	2.6	2.6
IPL	Outer inferior (I2)	27.38 ± 4.75	25.64 ± 4.84	0.142	0.641 (0.427, 0.855)	2.6	2.6
PPAA	RAT_23	0.261 ± 0.02	0.254 ± 0.02	0.136	0.628 (0.438, 0.818)	5.1	2.6

AUC: area under the receiver operating characteristic curve. BMO-MRW: Bruch’s membrane opening minimum rim width. CI: confidence interval. ETDRS: early treatment diabetic retinopathy. GCL: macular ganglion cell layer. IPL: macular inner plexiform layer. NFL: macular retinal nerve fiber layer. PPAA: posterior pole asymmetry analysis. RAT: retinal average thickness. RETINA: whole retinal layer. RNFL: circumpapillary retinal nerve fiber layer. *: Significance was set at *p* < 0.05.

**Table 6:** Optimal Combination of Parameters for Differentiating Between Individuals With and Without Disease Progression.

Parameters Included	AUC (95% CI)
Model 3I: Age, Refraction, MD, Hypertension, Diabetes mellitus	0.791 (0.624, 0.958)
Model 3: Age, PSD, MD, Diabetes mellitus, NFL (Outer inferior), RETINA (Inner inferior)	0.791 (0.624, 0.958)
Model 0: Age, Refraction, PSD, MD	0.715 (0.505, 0.925)

AUC: area under the receiver operating characteristic curve. CI: confidence interval. MD: mean deviation. NFL: macular retinal nerve fiber layer. PSD: pattern standard deviation. RETINA: whole retinal layer.

## Data Availability

The original contributions presented in this study are included in the article/Supplementary Material. Further inquiries can be directed to the corresponding author.

## Supplementary Materials

The following supporting information can be downloaded at: https://www.mdpi.com/article/10.3390/diagnostics16040534/s1, Table S1: Comparison of Patient Retinal Thickness Using the Optimal Parameters to Identify MCI; Table S2: Comparison of Patient Retinal Thickness Using the Optimal Parameters to Distinguish Patients With Mild Dementia From Healthy Controls; Table S3: Comparison of Patient Retinal Thickness Using the Optimal Parameters to Distinguish Patients With MCI From Those With Mild Dementia; Table S4: Comparison of Patient Retinal Thickness Using the Optimal Parameters to Distinguish Between Patients With and Without Disease Progression.
