# Supplementary material for: Early Detection of Dementia Through Spectralis Optical Coherence Tomography in a Taiwanese Cohort
Source: Diagnostics (Basel). 2026 Feb 11;16(4):534. doi: 10.3390/diagnostics16040534 (PMC12939327; doi:10.3390/diagnostics16040534)
Supplement: Supplementary file 1 [file diagnostics-16-00534-s001.zip › diagnostics-4067605-supplementary.pdf]

Table S1. Comparison of Patient Retinal Thickness Using the Optimal Parameters to Identify MCI

Scan	Parameter	Thickness (μm) (mean ± SD)		*P*	AUC	95% CI	Sensitivity at 95% specificity (%)	Sensitivity at 80% specificity (%)
		MCI	Control					
RNFL	T	80.02 ± 12.41	78.13 ± 18.53	0.340	0.524	0.390, 0.658	3.1	21.9
	TI	142.97 ± 20.92	149.45 ± 24.29	0.099	**0.404**	0.271, 0.536	21.9	28.1
	NI	107.28 ± 18.60	113.68 ± 30.14	0.123	0.423	0.289, 0.557	12.5	25.0
	N	77.63 ± 10.65	75.03 ± 14.24	0.188	0.591	0.457, 0.724	6.3	18.8
	NS	124.01 ± 20.19	116.95 ± 22.78	0.080	0.603	0.469, 0.736	9.4	18.8
	TS	136.12 ± 19.82	141.95 ± 19.61	0.116	**0.415**	0.284, 0.547	28.1	9.4
	G	100.33 ± 9.27	100.32 ± 13.65	0.479	**0.488**	0.351, 0.625	18.8	12.5
MRW	T	189.80 ± 31.98	206.87 ± 47.05	0.034	0.602	0.468, 0.737	0.0	2.3
	TI	288.33 ± 55.51	318.48 ± 43.93	0.004	**0.676**	0.554, 0.797	2.3	14.0
	NI	309.28 ± 58.39	354.19 ± 54.93	< 0.001	**0.720**	0.604, 0.836	2.3	7.0
	N	277.36 ± 50.48	306.34 ± 45.70	0.004	0.682	0.559, 0.804	2.3	11.6
	NS	313.64 ± 63.07	330.37 ± 50.23	0.097	0.604	0.474, 0.773	9.3	14.0
	TS	265.00 ± 49.54	293.06 ± 47.27	0.009	0.680	0.555, 0.805	3.1	11.6
	G	263.13 ± 40.76	286.69 ± 39.91	0.002	**0.675**	0.555, 0.796	0.0	9.3
ETDRS RETINA	T1	326.10 ± 35.43	318.98 ± 19.89	0.146	0.524	0.387, 0.660	11.6	14.0
	T2	278.91 ± 19.77	273.89 ± 15.07	0.147	0.463	0.328, 0.598	4.7	25.6
	I1	329.57 ± 23.22	327.42 ± 22.04	0.363	0.596	0.427, 0.711	7.0	11.6
	I2	275.70 ± 16.32	279.19 ± 17.73	0.154	0.606	0.471, 0.742	2.3	14.0
	N1	336.60 ± 29.45	330.37 ± 21.89	0.168	0.532	0.393, 0.671	14.0	20.9
	N2	306.02 ± 20.20	303.68 ± 21.89	0.369	0.487	0.352, 0.621	7.0	16.3
	S1	335.79 ± 32.95	327.11 ± 20.38	0.097	0.496	0.356, 0.636	11.6	11.6
	S2	293.08 ± 23.33	287.66 ± 16.44	0.155	0.515	0.376, 0.655	11.6	20.9
	C	277.83 ± 52.97	268.61 ± 21.61	0.144	0.524	0.392, 0.655	11.6	23.3

NFL	T1	21.00 ± 11.97	18.35 ± 2.81	0.087	0.515	0.383, 0.647	0.0	15.6
	T2	22.51 ± 5.00	20.87 ± 2.38	0.043	0.538	0.407, 0.669	0.0	9.4
	I1	28.22 ± 10.97	25.03 ± 4.95	0.049	0.566	0.436, 0.697	12.5	31.3
	I2	38.17 ± 6.98	39.47 ± 7.57	0.160	**0.449**	0.316, 0.582	0.0	3.1
	N1	24.70 ± 13.42	20.21 ± 2.46	0.019	0.610	0.483, 0.738	3.1	9.4
	N2	49.05 ± 10.29	44.87 ± 9.79	0.055	**0.578**	0.448, 0.709	0.0	3.1
	S1	27.52 ± 12.73	23.31 ± 3.34	0.025	0.573	0.443, 0.703	0.0	3.1
	S2	41.51 ± 15.26	37.79 ± 5.70	0.097	**0.518**	0.382, 0.653	0.0	6.3
	C	14.61 ± 10.46	12.10 ± 3.31	0.072	0.564	0.433, 0.695	0.0	9.4
GCL	T1	45.36 ± 5.68	43.21 ± 8.65	0.122	**0.524**	0.389, 0.660	3.1	6.3
	T2	34.02 ± 4.31	32.94 ± 4.41	0.245	**0.530**	0.393, 0.667	9.4	18.8
	I1	48.95 ± 6.99	47.39 ± 8.32	0.225	0.481	0.345, 0.617	3.1	28.1
	I2	31.17 ± 5.80	31.74 ± 4.31	0.263	0.416	0.283, 0.549	6.3	40.6
	N1	48.36 ± 5.41	45.53 ± 8.61	0.064	0.552	0.415, 0.689	0.0	31.3
	N2	37.77 ± 4.12	37.32 ± 4.86	0.390	0.495	0.360, 0.630	3.1	18.8
	S1	49.97 ± 6.02	47.79 ± 8.05	0.127	0.511	0.372, 0.649	3.1	31.3
	S2	34.49 ± 3.74	33.65 ± 4.01	0.232	**0.514**	0.377, 0.651	3.1	31.3
	C	17.38 ± 9.58	14.53 ± 5.46	0.050	0.585	0.454, 0.716	0.0	12.5
IPL	T1	41.15 ± 6.04	39.37 ±4.94	0.096	**0.522**	0.385, 0.659	14.0	18.6
	T2	32.06 ± 3.42	31.45 ± 3.22	0.307	**0.487**	0.350, 0.624	4.7	16.3
	I1	38.65 ± 3.87	38.65 ± 5.04	0.457	0.568	0.429, 0.706	4.7	11.6
	I2	26.09 ± 4.84	26.79 ± 3.97	0.214	0.584	0.451, 0.716	2.3	11.6
	N1	40.52 ± 3.51	39.21 ± 5.21	0.136	0.512	0.369, 0.656	11.6	14.0
	N2	29.65 ± 3.16	29.56 ± 3.49	0.466	0.527	0.393, 0.662	4.7	25.6
	S1	40.08 ± 5.52	38.42 ± 4.88	0.100	0.502	0.362, 0.642	7.0	11.6
	S2	28.24 ± 3.16	27.47 ± 2.99	0.185	**0.460**	0.325, 0.595	9.3	20.9
	C	21.48 ± 7.77	19.32 ± 3.67	0.053	0.450	0.318, 0.581	14.0	30.2
PPAA	RAT_11	0.225 ± 0.02	0.228 ± 0.01	0.232	0.579	0.446, 0.712	14.0	27.9
	RAT_12	0.232 ± 0.02	0.234 ± 0.01	0.262	0.573	0.441, 0.705	11.6	18.6
	RAT_13	0.242 ± 0.02	0.247 ± 0.02	0.109	0.601	0.470, 0.731	7.0	18.6

RAT_14	0.252 ± 0.02	0.260 ± 0.02	0.032	0.627	0.496, 0.758	2.3	11.6
RAT_15	0.262 ± 0.02	0.270 ± 0.02	0.024	0.639	0.508, 0.770	2.3	14.0
RAT_16	0.274 ± 0.02	0.285 ± 0.02	0.014	0.653	0.525, 0.781	2.3	4.7
RAT_17	0.283 ± 0.02	0.290 ± 0.02	0.058	0.595	0.465, 0.726	2.3	14.0
RAT_18	0.276 ± 0.02	0.279 ± 0.02	0.203	0.554	0.422, 0.686	4.7	18.6
RAT_21	0.227 ± 0.01	0.229 ± 0.01	0.240	0.590	0.458, 0.722	14.0	18.6
RAT_22	0.239 ± 0.02	0.243 ± 0.01	0.099	0.617	0.485, 0.749	9.3	18.6
RAT_23	0.256 ± 0.02	0.261 ± 0.02	0.063	0.622	0.490, 0.754	2.3	14.0
RAT_24	0.274 ± 0.02	0.278 ± 0.02	0.106	0.618	0.482, 0.753	2.3	4.7
RAT_25	0.280 ± 0.02	0.285 ± 0.02	0.111	0.624	0.488, 0.760	2.3	9.3
RAT_26	0.281 ± 0.02	0.285 ± 0.02	0.204	0.563	0.427, 0.699	2.3	16.3
RAT_27	0.291 ± 0.02	0.294 ± 0.03	0.250	0.549	0.414, 0.683	0.0	16.3
RAT_28	0.304 ± 0.02	0.309 ± 0.02	0.191	0.546	0.413, 0.679	4.7	18.6
RAT_31	0.239 ± 0.01	0.241 ± 0.01	0.231	0.560	0.425, 0.696	2.3	23.3
RAT_32	0.262 ± 0.02	0.263 ± 0.02	0.335	0.572	0.436, 0.708	7.0	16.3
RAT_33	0.295 ± 0.02	0.296 ± 0.02	0.318	0.585	0.447, 0.724	4.7	9.3
RAT_34	0.321 ± 0.02	0.322 ± 0.02	0.402	0.601	0.457, 0.745	4.7	9.3
RAT_35	0.327 ± 0.02	0.325 ± 0.02	0.451	0.580	0.439, 0.721	4.7	9.3
RAT_36	0.316 ± 0.02	0.314 ± 0.02	0.430	0.516	0.378, 0.654	2.3	16.3
RAT_37	0.298 ± 0.02	0.298 ± 0.02	0.467	0.497	0.360, 0.634	4.7	20.9
RAT_38	0.305 ± 0.02	0.307 ± 0.03	0.357	0.502	0.367, 0.638	0.0	14.0
RAT_41	0.249 ± 0.02	0.253 ± 0.02	0.150	0.601	0.470, 0.733	2.3	7.0
RAT_42	0.281 ± 0.03	0.282 ± 0.02	0.325	0.591	0.457, 0.725	4.7	14.0
RAT_43	0.321 ± 0.03	0.319 ± 0.02	0.414	0.554	0.416, 0.691	4.7	11.6
RAT_44	0.312 ± 0.04	0.308 ± 0.02	0.279	0.530	0.396, 0.665	7.0	16.3
RAT_45	0.312 ± 0.04	0.307 ± 0.02	0.236	0.532	0.398, 0.665	9.3	20.9
RAT_46	0.339 ± 0.02	0.334 ± 0.02	0.199	0.511	0.372, 0.649	11.6	16.3
RAT_47	0.314 ± 0.02	0.308 ± 0.02	0.156	0.446	0.313, 0.580	9.3	25.6
RAT_48	0.291 ± 0.02	0.285 ± 0.02	0.106	0.430	0.298, 0.561	9.3	30.2
RAT_51	0.247 ± 0.01	0.246 ± 0.01	0.409	0.506	0.371, 0.641	6.3	28.1

RAT_52	0.281 ± 0.03	0.275 ± 0.02	0.153	0.523	0.386, 0.660	0.0	25.0
RAT_53	0.321 ± 0.04	0.312 ± 0.02	0.100	0.513	0.375, 0.651	0.0	28.1
RAT_54	0.311 ± 0.04	0.304 ± 0.02	0.156	0.488	0.355, 0.621	0.0	21.9
RAT_55	0.315 ± 0.04	0.308 ± 0.02	0.180	0.469	0.334, 0.603	0.0	21.9
RAT_56	0.342 ± 0.03	0.334 ± 0.02	0.084	0.528	0.389, 0.667	0.0	18.8
RAT_57	0.318 ± 0.02	0.308 ± 0.02	0.042	0.593	0.459, 0.727	0.0	18.8
RAT_58	0.293 ± 0.02	0.285 ± 0.02	0.044	0.593	0.462, 0.724	0.0	15.6
RAT_61	0.243 ± 0.02	0.242 ± 0.01	0.430	0.480	0.346, 0.614	0.0	25.0
RAT_62	0.267 ± 0.02	0.263 ± 0.01	0.187	0.527	0.393, 0.661	0.0	21.9
RAT_63	0.299 ± 0.02	0.292 ± 0.02	0.086	0.521	0.383, 0.660	0.0	25.0
RAT_64	0.327 ± 0.03	0.319 ± 0.02	0.078	0.494	0.353, 0.635	0.0	37.5
RAT_65	0.335 ± 0.03	0.328 ± 0.02	0.414	0.489	0.347, 0.631	3.1	37.5
RAT_66	0.323 ± 0.03	0.316 ± 0.02	0.136	0.512	0.370, 0.654	0.0	40.6
RAT_67	0.306 ± 0.02	0.300 ± 0.02	0.097	0.566	0.430, 0.7002	3.1	21.9
RAT_68	0.304 ± 0.02	0.299 ± 0.02	0.184	0.528	0.393, 0.663	0.0	12.5
RAT_71	0.234 ± 0.02	0.235 ± 0.01	0.373	0.579	0.447, 0.711	14.0	20.9
RAT_72	0.248 ± 0.02	0.248 ± 0.02	0.395	0.553	0.417, 0.688	7.0	11.6
RAT_73	0.265 ± 0.01	0.265 ± 0.02	0.391	0.556	0.416, 0.696	7.0	11.6
RAT_74	0.286 ± 0.02	0.283 ± 0.02	0.284	0.513	0.375, 0.651	9.3	18.6
RAT_75	0.297 ± 0.02	0.294 ± 0.02	0.283	0.530	0.390, 0.670	11.6	16.3
RAT_76	0.293 ± 0.02	0.291 ± 0.02	0.384	0.545	0.405, 0.685	9.3	11..6
RAT_77	0.290 ± 0.02	0.289 ± 0.02	0.496	0.535	0.397, 0.672	9.3	16.3
RAT_78	0.301 ± 0.02	0.303 ± 0.02	0.319	0.559	0.424, 0.693	4.7	16.3
RAT_81	0.226 ± 0.01	0.229 ± 0.02	0.141	0.594	0.459, 0.728	2.3	14.0
RAT_82	0.233 ± 0.01	0.236 ± 0.01	0.074	0.647	0.512, 0.781	2.3	11.6
RAT_83	0.245 ± 0.01	0.247 ± 0.01	0.147	0.602	0.464, 0.741	2.3	7.0
RAT_84	0.258 ± 0.01	0.259 ± 0.01	0.375	0.559	0.423, 0.695	7.0	14.0
RAT_85	0.269 ± 0.02	0.270 ± 0.02	0.277	0.592	0.456, 0.729	4.7	14.0
RAT_86	0.274 ± 0.02	0.277 ± 0.02	0.197	0.608	0.472, 0.744	4.7	9.3
RAT_87	0.281 ± 0.02	0.286 ± 0.02	0.075	0.634	0.504, 0.765	4.7	11.6

	RAT_88	0.296 ± 0.02	0.301 ± 0.02	0.128	0.597	0.469, 0.726	2.3	18.6

AUC: area under the receiver operating characteristic curve. C: central. CI: confidence interval. ETDRS: early treatment of diabetic retinopathy study G: global. GCL: macular ganglion cell layer. I1: inner inferior. I2: outer inferior. IPL: macular inner plexiform layer. MRW: Bruch's membrane opening minimum rim width. N: nasal. N1: inner nasal. N2: outer nasal. NFL: macular retinal nerve fiber layer. NI: nasal inferior. NS: nasal superior. PPAA: posterior pole asymmetry analysis. RAT: retinal average thickness. RETINA: whole retinal layer. RNFL: circumpapillary retinal nerve fiber layer. S1: inner superior. S2: outer superior. T: temporal. T1: inner temporal. T2: outer temporal. TI: temporal inferior. TS: temporal superior.

Table S2. Comparison of Patient Retinal Thickness Using the Optimal Parameters to Distinguish Patients With Mild Dementia From Healthy Controls

Scan	Parameter	Thickness (μm) (mean ± SD)		*P*	AUC	95% CI	Sensitivity at 95% specificity (%)	Sensitivity at 80% specificity (%)
		Mild dementia	Control					
RNFL	T	84.08 ± 39.78	78.13 ± 18.53	0.317	0.659	0.467, 0.850	15.4	15.4
	TI	159.27 ± 23.96	149.45 ± 24.29	0.123	**0.419**	0.243, 0.596	7.7	15.4
	NI	144.46 ± 21.31	113.68 ± 30.14	0.493	0.472	0.283, 0.662	0.0	15.4
	N	81.54 ± 12.11	75.03 ± 14.24	0.064	0.355	0.181, 0.528	15.4	46.2
	NS	120.65 ± 18.37	116.95 ± 22.78	0.282	0.428	0.253, 0.603	7.7	30.8
	TS	138.77 ± 21.59	141.95 ± 19.61	0.342	**0.505**	0.322, 0.688	0.0	7.7
	G	103.01 ± 12.48	100.32 ± 13.65	0.273	**0.459**	0.270, 0.648	7.7	38.5
MRW	T	172.08 ± 23.28	206.87 ± 47.05	0.001	0.725	0.576, 0.873	0.0	0.0
	TI	300.12 ± 29.87	318.48 ± 43.93	0.050	**0.636**	0.469, 0.802	0.0	7.7
	NI	331.88 ± 48.30	354.19 ± 54.93	0.080	**0.632**	0.449, 0.816	0.0	7.7
	N	284.31 ± 48.49	306.34 ± 45.70	0.076	0.620	0.431, 0.809	0.0	7.7
	NS	300.15 ± 65.31	330.37 ± 50.23	0.073	0.660	0.478, 0.842	7.7	7.7
	TS	246.85 ± 40.02	293.06 ± 47.27	0.002	0.764	0.623, 0.905	0.0	0.0
	G	261.35 ± 31.20	286.69 ± 39.91	0.007	**0.707**	0.547, 0.866	0.0	7.7
ETDRS RETINA	T1	317.23 ± 34.24	318.98 ± 19.89	0.421	0.653	0.477, 0.828	7.7	7.7
	T2	268.54 ± 19.63	273.89 ± 15.07	0.166	0.643	0.457, 0.89	7.7	15.4
	I1	323.42 ± 32.76	327.42 ± 22.04	0.334	0.667	0.494, 0.840	7.7	7.7
	I2	274.50 ± 22.68	279.19 ± 17.73	0.230	0.421	0.424, 0.818	15.4	23.1
	N1	326.88 ± 37.16	330.37 ± 21.89	0.360	0.665	0.485, 0.844	7.7	15.4
	N2	305.15 ± 22.09	303.68 ± 21.89	0.458	0.526	0.342, 0.711	7.7	7.7
	S1	326.58 ± 31.54	327.11 ± 20.38	0.454	0.609	0.432, 0.787	7.7	7.7

	S2	287.88 ± 19.99	287.66 ± 16.44	0.470	0.553	0.365, 0.741	7.7	23.1
	C	273.35 ± 65.13	268.61 ± 21.61	0.394	0.648	0.428, 0.868	15.4	23.1
NFL	T1	20.54 ± 9.15	18.35 ± 2.81	0.210	0.481	0.291, 0.671	7.7	23.1
	T2	21.19 ± 3.73	20.87 ± 2.38	0.428	0.558	0.360, 0.756	7.7	23.1
	I1	27.04 ± 12.19	25.03 ± 4.95	0.290	0.541	0.363, 0.718	7.7	15.4
	I2	40.50 ± 9.68	39.47 ± 7.57	0.427	**0.518**	0.330, 0.706	7.7	23.1
	N1	22.35 ± 11.60	20.21 ± 2.46	0.264	0.583	0.392, 0.774	7.7	15.4
	N2	45.69 ± 12.49	44.87 ± 9.79	0.455	**0.597**	0.418, 0.777	7.7	15.4
	S1	25.04 ± 9.48	23.31 ± 3.34	0.283	0.567	0.384, 0.750	7.7	23.1
	S2	39.04 ± 9.11	37.79 ± 5.70	0.376	**0.546**	0.359, 0.732	7.7	23.1
	C	15.58 ± 15.40	12.10 ± 3.31	0.217	0.569	0.357, 0.780	7.7	30.8
GCL	T1	44.54 ± 6.95	43.21 ± 8.65	0.310	**0.578**	0.404, 0.752	7.7	7.7
	T2	32.35 ± 5.19	32.94 ± 4.41	0.290	**0.570**	0.371, 0.769	7.7	30.8
	I1	48.31 ± 7.36	47.39 ± 8.32	0.387	0.560	0.378, 0.742	7.7	15.4
	I2	30.69 ± 5.09	31.74 ± 4.31	0.227	0.606	0.416, 0.796	7.7	23.1
	N1	44.65 ± 8.86	45.53 ± 8.61	0.360	0.633	0.444, 0.823	7.7	15.4
	N2	38.08 ± 4.05	37.32 ± 4.86	0.335	0.499	0.314, 0.683	15.4	15.4
	S1	48.58 ± 8.02	47.79 ± 8.05	0.417	0.593	0.418, 0.767	7.7	7.7
	S2	33.69 ± 4.57	33.65 ± 4.01	0.468	**0.508**	0.308, 0.709	7.7	30.8
	C	19.00 ± 14.82	14.53 ± 5.46	0.152	0.531	0.318, 0.744	15.4	30.8
IPL	T1	40.31 ± 6.55	39.37 ±4.94	0.344	**0.570**	0.390, 0.750	7.7	7.7
	T2	30.85 ± 4.43	31.45 ± 3.22	0.280	**0.589**	0.398, 0.779	7.7	15.4
	I1	39.12 ± 5.92	38.65 ± 5.04	0.427	0.597	0.423, 0.772	7.7	7.7
	I2	25.73 ± 4.02	26.79 ± 3.97	0.191	0.626	0.438, 0.815	0.0	15.4
	N1	39.31 ± 8.09	39.21 ± 5.21	0.494	0.620	0.430, 0.811	7.7	15.4
	N2	30.31 ± 3.44	29.56 ± 3.49	0.305	0.507	0.320, 0.694	7.7	23.1
	S1	38.69 ± 6.31	38.42 ± 4.88	0.467	0.608	0.431, 0.785	7.7	7.7
	S2	27.92 ± 3.37	27.47 ± 2.99	0.381	**0.488**	0.291, 0.685	7.7	38.5
	C	23.50 ± 12.87	19.32 ± 3.67	0.132	0.525	0.310, 0.740	23.1	30.8
PPAA	RAT_11	0.233 ± 0.02	0.228 ± 0.01	0.213	0.450	0.232, 0.667	23.1	46.2

RAT_12	0.235 ± 0.02	0.234 ± 0.01	0.485	0.499	0.273, 0.725	23.1	46.2
RAT_13	0.242 ± 0.02	0.247 ± 0.02	0.211	0.575	0.375, 0.774	7.7	30.8
RAT_14	0.253 ± 0.02	0.260 ± 0.02	0.124	0.612	0.423, 0.800	0.0	15.4
RAT_15	0.265 ± 0.02	0.270 ± 0.02	0.208	0.583	0.391, 0.775	7.7	23.1
RAT_16	0.281 ± 0.02	0.285 ± 0.02	0.292	0.556	0.365, 0.748	7.7	23.1
RAT_17	0.293 ± 0.02	0.290 ± 0.02	0.354	0.493	0.283, 0.703	23.1	38.5
RAT_18	0.281 ± 0.02	0.279 ± 0.02	0.429	0.511	0.300, 0.722	23.1	30.8
RAT_21	0.229 ± 0.01	0.229 ± 0.01	0.478	0.511	0.306, 0.716	23.1	30.8
RAT_22	0.240 ± 0.02	0.243 ± 0.01	0.273	0.588	0.387, 0.789	15.4	23.1
RAT_23	0.256 ± 0.02	0.261 ± 0.02	0.172	0.611	0.409, 0.812	15.4	23.1
RAT_24	0.274 ± 0.02	0.278 ± 0.02	0.265	0.593	0.396, 0.789	15.4	23.1
RAT_25	0.283 ± 0.03	0.285 ± 0.02	0.395	0.571	0.376, 0.766	15.4	23.1
RAT_26	0.284 ± 0.03	0.285 ± 0.02	0.436	0.553	0.356, 0.750	15.4	23.1
RAT_27	0.295 ± 0.03	0.294 ± 0.03	0.477	0.536	0.339, 0.734	15.4	23.1
RAT_28	0.311 ± 0.02	0.309 ± 0.02	0.381	0.463	0.266, 0.660	7.7	30.8
RAT_31	0.240 ± 0.02	0.241 ± 0.01	0.409	0.630	0.456, 0.804	7.7	7.7
RAT_32	0.261 ± 0.03	0.263 ± 0.02	0.354	0.630	0.433, 0.827	7.7	23.1
RAT_33	0.291 ± 0.03	0.296 ± 0.02	0.274	0.633	0.430, 0.837	7.7	23.1
RAT_34	0.318 ± 0.03	0.322 ± 0.02	0.329	0.642	0.456, 0.827	7.7	15.4
RAT_35	0.322 ± 0.03	0.325 ± 0.02	0.349	0.650	0.471, 0.829	7.7	7.7
RAT_36	0.311 ± 0.02	0.314 ± 0.02	0.307	0.588	0.403, 0.773	7.7	15.4
RAT_37	0.295 ± 0.02	0.298 ± 0.02	0.359	0.564	0.374, 0.753	7.7	23.1
RAT_38	0.301 ± 0.02	0.307 ± 0.03	0.223	0.544	0.352, 0.736	7.7	15.4
RAT_41	0.244 ± 0.02	0.253 ± 0.02	0.080	0.708	0.528, 0.888	7.7	15.4
RAT_42	0.275 ± 0.03	0.282 ± 0.02	0.210	0.691	0.506, 0.876	7.7	15.4
RAT_43	0.314 ± 0.04	0.319 ± 0.02	0.315	0.684	0.504, 0.864	7.7	15.4
RAT_44	0.309 ± 0.05	0.308 ± 0.02	0.472	0.605	0.414, 0.795	7.7	15.4
RAT_45	0.302 ± 0.05	0.307 ± 0.02	0.351	0.639	0.442, 0.837	7.7	15.4
RAT_46	0.332 ± 0.02	0.334 ± 0.02	0.345	0.632	0.453, 0.812	7.7	15.4
RAT_47	0.310 ± 0.02	0.308 ± 0.02	0.439	0.506	0.323, 0.689	7.7	23.1

RAT_48	0.286 ± 0.02	0.285 ± 0.02	0.454	0.502	0.307, 0.697	7.7	23.1
RAT_51	0.247 ± 0.02	0.246 ± 0.01	0.441	0.536	0.329, 0.743	7.7	30.8
RAT_52	0.273 ± 0.03	0.275 ± 0.02	0.365	0.613	0.422, 0.804	7.7	15.4
RAT_53	0.311 ± 0.03	0.312 ± 0.02	0.454	0.629	0.446, 0.812	7.7	15.4
RAT_54	0.307 ± 0.04	0.304 ± 0.02	0.401	0.569	0.377, 0.760	7.7	15.4
RAT_55	0.303 ± 0.04	0.308 ± 0.02	0.338	0.660	0.465, 0.855	7.7	15.4
RAT_56	0.332 ± 0.02	0.334 ± 0.02	0.369	0.602	0.423, 0.782	7.7	7.7
RAT_57	0.314 ± 0.02	0.308 ± 0.02	0.271	0.466	0.286, 0.647	7.7	30.8
RAT_58	0.288 ± 0.02	0.285 ± 0.02	0.270	0.453	0.270, 0.637	7.7	30.8
RAT_61	0.240 ± 0.02	0.242 ± 0.01	0.280	0.577	0.392, 0.762	7.7	15.4
RAT_62	0.262 ± 0.02	0.263 ± 0.01	0.433	0.561	0.378, 0.744	7.7	15.4
RAT_63	0.293 ± 0.03	0.292 ± 0.02	0.482	0.589	0.405, 0.773	7.7	15.4
RAT_64	0.320 ± 0.03	0.319 ± 0.02	0.487	0.613	0.427, 0.799	7.7	15.4
RAT_65	0.326 ± 0.02	0.328 ± 0.02	0.355	0.595	0.416, 0.773	7.7	7.7
RAT_66	0.314 ± 0.02	0.316 ± 0.02	0.364	0.538	0.353, 0.724	7.7	15.4
RAT_67	0.299 ± 0.02	0.300 ± 0.02	0.408	0.544	0.355, 0.734	7.7	15.4
RAT_68	0.295 ± 0.01	0.299 ± 0.02	0.200	0.548	0.372, 0.724	0.0	7.7
RAT_71	0.234 ± 0.01	0.235 ± 0.01	0.345	0.594	0.404, 0.783	7.7	15.4
RAT_72	0.246 ± 0.01	0.248 ± 0.02	0.277	0.594	0.414, 0.773	7.7	7.7
RAT_73	0.265 ± 0.02	0.265 ± 0.02	0.414	0.599	0.419, 0.779	7.7	15.4
RAT_74	0.284 ± 0.03	0.283 ± 0.02	0.470	0.589	0.402, 0.776	7.7	15.4
RAT_75	0.294 ± 0.02	0.294 ± 0.02	0.467	0.531	0.339, 0.724	7.7	30.8
RAT_76	0.290 ± 0.02	0.291 ± 0.02	0.379	0.549	0.356, 0.743	15.4	23.1
RAT_77	0.288 ± 0.02	0.289 ± 0.02	0.411	0.548	0.364, 0.732	7.7	15.4
RAT_78	0.298 ± 0.02	0.303 ± 0.02	0.215	0.582	0.403, 0.761	7.7	15.4
RAT_81	0.233 ± 0.02	0.229 ± 0.02	0.303	0.520	0.311, 0.730	23.1	30.8
RAT_82	0.236 ± 0.01	0.236 ± 0.01	0.485	0.581	0.386, 0.775	7.7	15.4
RAT_83	0.248 ± 0.01	0.247 ± 0.01	0.470	0.561	0.382, 0.740	7.7	7.7
RAT_84	0.260 ± 0.01	0.259 ± 0.01	0.473	0.552	0.371, 0.732	7.7	15.4
RAT_85	0.272 ± 0.01	0.270 ± 0.02	0.451	0.544	0.360, 0.729	7.7	15.4

RAT_86	0.277 ± 0.02	0.277 ± 0.02	0.466	0.542	0.354, 0.730	15.4	15.4
RAT_87	0.274 ± 0.01	0.286 ± 0.02	0.315	0.554	0.372, 0.736	0.0	15.4
RAT_88	0.297 ± 0.02	0.301 ± 0.02	0.233	0.575	0.380, 0.769	0.0	23.1

AUC: area under the receiver operating characteristic curve. C: central. CI: confidence interval. ETDRS: early treatment diabetic retinopathy study. G: global. GCL: macular ganglion cell layer. I1: inner inferior. I2: outer inferior. IPL: macular inner plexiform layer. MRW: Bruch's membrane opening minimum rim width. N: nasal. N1: inner nasal. N2: outer nasal. NFL: macular retinal nerve fiber layer. NI: nasal inferior. NS: nasal superior. PPAA: posterior pole asymmetry analysis. RAT: retinal average thickness. RETINA: whole retinal layer. RNFL: circumpapillary retinal nerve fiber layer. S1: inner superior. S2: outer superior. T: temporal. T1: inner temporal. T2: outer temporal. TI: temporal inferior. TS: temporal superior.

Table S3. Comparison of Patient Retinal Thickness Using the Optimal Parameters to Distinguish Patients With MCI From Those With Mild Dementia

Scan	Parameter	Thickness (μm) (mean ± SD)		*P*	AUC	95% CI	Sensitivity at 95% specificity (%)	Sensitivity at 80% specificity (%)
		MCI	Mild dementia					
RNFL	T	80.02 ± 12.41	84.08 ± 39.78	0.362	0.316	0.127, 0.504	0.0	46.5
	TI	142.97 ± 20.92	159.27 ± 23.96	0.020	**0.695**	0.531, 0.859	0.0	4.7
	NI	107.28 ± 18.60	144.46 ± 21.31	0.144	0.587	0.399, 0.774	2.3	14.0
	N	77.63 ± 10.65	81.54 ± 12.11	0.154	0.583	0.392, 0.774	2.3	7.0
	NS	124.01 ± 20.19	120.65 ± 18.37	0.289	0.453	0.268, 0.639	2.3	27.9
	TS	136.12 ± 19.82	138.77 ± 21.59	0.349	**0.586**	0.406, 0.766	11.6	16.3
	G	100.33 ± 9.27	103.01 ± 12.48	0.239	**0.543**	0.355, 0.731	0.0	18.6
MRW	T	189.80 ± 31.98	172.08 ± 23.28	0.019	0.659	0.508, 0.810	0.0	0.0
	TI	288.33 ± 55.51	300.12 ± 29.87	0.163	**0.406**	0.255, 0.558	0.0	15.4
	NI	309.28 ± 58.39	331.88 ± 48.30	0.086	**0.382**	0.222, 0.542	7.7	23.1
	N	277.36 ± 50.48	284.31 ± 48.49	0.329	0.460	0.277, 0.642	0.0	38.5
	NS	313.64 ± 63.07	300.15 ± 65.31	0.259	0.560	0.383, 0.737	7.7	23.1
	TS	265.00 ± 49.54	246.85 ± 40.02	0.094	0.589	0.420, 0.757	0.0	7.7
	G	263.13 ± 40.76	261.35 ± 31.20	0.434	**0.496**	0.331, 0.661	0.0	7.7
ETDRS RETINA	T1	326.10 ± 35.43	317.23 ± 34.24	0.213	0.639	0.468, 0.809	7.7	7.7
	T2	278.91 ± 19.77	268.54 ± 19.63	0.056	0.707	0.528, 0.885	7.7	15.4
	I1	329.57 ± 23.22	323.42 ± 32.76	0.269	0.679	0.497, 0.860	7.7	15.4
	I2	275.70 ± 16.32	274.50 ± 22.68	0.431	0.559	0.361, 0.757	15.4	23.1
	N1	336.60 ± 29.45	326.88 ± 37.16	0.200	0.699	0.511, 0.888	7.7	15.4
	N2	306.02 ± 20.20	305.15 ± 22.09	0.450	0.563	0.380, 0.745	7.7	23.1
	S1	335.79 ± 32.95	326.58 ± 31.54	0.186	0.683	0.511, 0.856	7.7	7.7

	S2	293.08 ± 23.33	287.88 ± 19.99	0.219	0.593	0.393, 0.773	7.7	23.1
	C	277.83 ± 52.97	273.35 ± 65.13	0.412	0.621	0.422, 0.820	7.7	23.1
NFL	T1	21.00 ± 11.97	20.54 ± 9.15	0.442	0.494	0.320, 0.667	7.7	23.1
	T2	22.51 ± 5.00	21.19 ± 3.73	0.157	0.590	0.416, 0.765	0.0	15.4
	I1	28.22 ± 10.97	27.04 ± 12.19	0.379	0.604	0.441, 0.766	7.7	7.7
	I2	38.17 ± 6.98	40.50 ± 9.68	0.216	**0.461**	0.277, 0.644	7.7	30.8
	N1	24.70 ± 13.42	22.35 ± 11.60	0.272	0.662	0.494, 0.830	7.7	7.7
	N2	49.05 ± 10.29	45.69 ± 12.49	0.195	**0.659**	0.493, 0.825	7.7	7.7
	S1	27.52 ± 12.73	25.04 ± 9.48	0.227	0.630	0.466, 0.793	0.0	7.7
	S2	41.51 ± 15.26	39.04 ± 9.11	0.238	**0.555**	0.373, 0.738	7.7	7.7
	C	14.61 ± 10.46	15.58 ± 15.40	0.418	0.617	0.410, 0.824	7.7	30.8
GCL	T1	45.36 ± 5.68	44.54 ± 6.95	0.351	**0.564**	0.393, 0.736	7.7	7.7
	T2	34.02 ± 4.31	32.35 ± 5.19	0.152	**0.605**	0.404, 0.805	7.7	23.1
	I1	48.95 ± 6.99	48.31 ± 7.36	0.391	0.564	0.391, 0.746	7.7	15.4
	I2	31.17 ± 5.80	30.69 ± 5.09	0.388	0.547	0.357, 0.736	7.7	23.1
	N1	48.36 ± 5.41	44.65 ± 8.86	0.087	0.723	0.532, 0.914	7.7	15.4
	N2	37.77 ± 4.12	38.08 ± 4.05	0.406	0.476	0.300, 0.652	15.4	15.4
	S1	49.97 ± 6.02	48.58 ± 8.02	0.286	0.631	0.454, 0.809	7.7	7.7
	S2	34.49 ± 3.74	33.69 ± 4.57	0.287	**0.538**	0.324, 0.751	15.4	30.8
	C	17.38 ± 9.58	19.00 ± 14.82	0.359	0.598	0.386, 0.810	15.4	23.1
IPL	T1	41.15 ± 6.04	40.31 ± 6.55	0.342	**0.524**	0.338, 0.710	7.7	15.4
	T2	32.06 ± 3.42	30.85 ± 4.43	0.188	**0.614**	0.420, 0.809	7.7	23.1
	I1	38.65 ± 3.87	39.12 ± 5.92	0.397	0.522	0.341, 0.704	7.7	15.4
	I2	26.09 ± 4.84	25.73 ± 4.02	0.394	0.534	0.346, 0.722	7.7	23.1
	N1	40.52 ± 3.51	39.31 ± 8.09	0.304	0.697	0.497, 0.897	7.7	23.1
	N2	29.65 ± 3.16	30.31 ± 3.44	0.273	0.462	0.289, 0.636	7.7	23.1
	S1	40.08 ± 5.52	38.69 ± 6.31	0.242	0.651	0.463, 0.839	7.7	7.7
	S2	28.24 ± 3.16	27.92 ± 3.37	0.382	**0.528**	0.322, 0.733	7.7	30.8
	C	21.48 ± 7.77	23.50 ± 12.87	0.299	0.561	0.355, 0.766	7.7	30.8
PPAA	RAT_11	0.225 ± 0.02	0.233 ± 0.02	0.122	0.588	0.403, 0.773	4.7	9.3

RAT_12	0.232 ± 0.02	0.235 ± 0.02	0.346	0.514	0.308, 0.720	2.3	11.6
RAT_13	0.242 ± 0.02	0.242 ± 0.02	0.469	0.496	0.301, 0.692	7.0	14.0
RAT_14	0.252 ± 0.02	0.253 ± 0.02	0.452	0.505	0.315, 0.696	4.7	11.6
RAT_15	0.262 ± 0.02	0.265 ± 0.02	0.330	0.528	0.334, 0.722	7.7	18.6
RAT_16	0.274 ± 0.02	0.281 ± 0.02	0.190	0.585	0.391, 0.779	0.0	4.7
RAT_17	0.283 ± 0.02	0.293 ± 0.02	0.087	0.599	0.410, 0.789	2.3	2.3
RAT_18	0.276 ± 0.02	0.281 ± 0.02	0.266	0.533	0.336, 0.730	4.7	4.7
RAT_21	0.227 ± 0.01	0.229 ± 0.01	0.294	0.572	0.392, 0.751	4.7	9.3
RAT_22	0.239 ± 0.02	0.240 ± 0.02	0.397	0.485	0.296, 0.673	2.3	18.6
RAT_23	0.256 ± 0.02	0.256 ± 0.02	0.493	0.485	0.285, 0.684	2.3	14.0
RAT_24	0.274 ± 0.02	0.274 ± 0.02	0.465	0.481	0.275, 0.687	2.3	4.7
RAT_25	0.280 ± 0.02	0.283 ± 0.03	0.342	0.498	0.297, 0.699	2.3	9.3
RAT_26	0.281 ± 0.02	0.284 ± 0.03	0.381	0.499	0.305, 0.693	2.3	11.6
RAT_27	0.291 ± 0.02	0.295 ± 0.03	0.300	0.514	0.321, 0.708	0.0	9.3
RAT_28	0.304 ± 0.02	0.311 ± 0.02	0.167	0.581	0.390, 0.772	0.0	7.0
RAT_31	0.239 ± 0.01	0.240 ± 0.02	0.439	0.568	0.396, 0.740	7.7	7.7
RAT_32	0.262 ± 0.02	0.261 ± 0.03	0.432	0.594	0.398, 0.789	7.7	23.1
RAT_33	0.295 ± 0.02	0.291 ± 0.03	0.354	0.643	0.438, 0.849	7.7	23.1
RAT_34	0.321 ± 0.02	0.318 ± 0.03	0.366	0.634	0.424, 0.844	7.7	30.8
RAT_35	0.327 ± 0.02	0.322 ± 0.03	0.317	0.639	0.453, 0.825	7.7	23.1
RAT_36	0.316 ± 0.02	0.311 ± 0.02	0.248	0.613	0.423, 0.802	7.7	23.1
RAT_37	0.298 ± 0.02	0.295 ± 0.02	0.368	0.577	0.385, 0.769	7.7	23.1
RAT_38	0.305 ± 0.02	0.301 ± 0.02	0.286	0.552	0.357, 0.747	7.7	23.1
RAT_41	0.249 ± 0.02	0.244 ± 0.02	0.217	0.654	0.467, 0.840	7.7	15.4
RAT_42	0.281 ± 0.03	0.275 ± 0.03	0.288	0.639	0.449, 0.828	7.7	15.4
RAT_43	0.321 ± 0.03	0.314 ± 0.04	0.284	0.687	0.505, 0.869	7.7	15.4
RAT_44	0.312 ± 0.04	0.309 ± 0.05	0.420	0.601	0.409, 0.793	7.7	15.4
RAT_45	0.312 ± 0.04	0.302 ± 0.05	0.241	0.632	0.434, 0.831	7.7	15.4
RAT_46	0.339 ± 0.02	0.332 ± 0.02	0.155	0.660	0.481, 0.840	7.7	15.4
RAT_47	0.314 ± 0.02	0.310 ± 0.02	0.261	0.581	0.399, 0.762	7.7	15.4

RAT_48	0.291 ± 0.02	0.286 ± 0.02	0.203	0.586	0.402, 0.769	0.0	15.4
RAT_51	0.247 ± 0.01	0.247 ± 0.02	0.484	0.561	0.352, 0.769	7.7	30.8
RAT_52	0.281 ± 0.03	0.273 ± 0.03	0.191	0.693	0.507, 0.880	7.7	15.4
RAT_53	0.321 ± 0.04	0.311 ± 0.03	0.204	0.691	0.516, 0.865	7.7	15.4
RAT_54	0.311 ± 0.04	0.307 ± 0.04	0.371	0.559	0.375, 0.743	7.7	15.4
RAT_55	0.315 ± 0.04	0.303 ± 0.04	0.191	0.663	0.464, 0.862	7.7	15.4
RAT_56	0.342 ± 0.03	0.332 ± 0.02	0.093	0.658	0.474, 0.842	7.7	7.7
RAT_57	0.318 ± 0.02	0.314 ± 0.02	0.260	0.588	0.398, 0.777	7.7	23.1
RAT_58	0.293 ± 0.02	0.288 ± 0.02	0.196	0.547	0.371, 0.724	0.0	23.1
RAT_61	0.243 ± 0.02	0.240 ± 0.02	0.242	0.570	0.386, 0.751	0.0	15.4
RAT_62	0.267 ± 0.02	0.262 ± 0.02	0.230	0.607	0.426, 0.789	7.7	15.4
RAT_63	0.299 ± 0.02	0.293 ± 0.03	0.261	0.682	0.502, 0.863	7.7	15.4
RAT_64	0.327 ± 0.03	0.320 ± 0.03	0.212	0.678	0.493, 0.863	7.7	15.4
RAT_65	0.335 ± 0.03	0.326 ± 0.02	0.120	0.650	0.456, 0.844	7.7	23.1
RAT_66	0.323 ± 0.03	0.314 ± 0.02	0.126	0.615	0.419, 0.812	7.7	23.1
RAT_67	0.306 ± 0.02	0.299 ± 0.02	0.138	0.627	0.439, 0.815	7.7	15.4
RAT_68	0.304 ± 0.02	0.295 ± 0.01	0.044	0.607	0.431, 0.783	0.0	0.0
RAT_71	0.234 ± 0.02	0.234 ± 0.01	0.452	0.496	0.322, 0.669	7.7	15.4
RAT_72	0.248 ± 0.02	0.246 ± 0.01	0.354	0.507	0.338, 0.676	7.7	15.4
RAT_73	0.265 ± 0.01	0.265 ± 0.02	0.477	0.534	0.361, 0.725	7.7	15.4
RAT_74	0.286 ± 0.02	0.284 ± 0.03	0.355	0.625	0.433, 0.817	7.7	15.4
RAT_75	0.297 ± 0.02	0.294 ± 0.02	0.329	0.573	0.369, 0.778	7.7	30.8
RAT_76	0.293 ± 0.02	0.290 ± 0.02	0.309	0.555	0.356, 0.753	7.7	30.8
RAT_77	0.290 ± 0.02	0.288 ± 0.02	0.413	0.541	0.350, 0.733	7.7	30.8
RAT_78	0.301 ± 0.02	0.298 ± 0.02	0.328	0.522	0.346, 0.698	7.7	15.4
RAT_81	0.226 ± 0.01	0.233 ± 0.02	0.151	0.518	0.303, 0.733	0.0	2.3
RAT_82	0.233 ± 0.01	0.236 ± 0.01	0.194	0.542	0.353, 0.731	2.3	11.6
RAT_83	0.245 ± 0.01	0.248 ± 0.01	0.254	0.511	0.318, 0.703	2.3	14.0
RAT_84	0.258 ± 0.01	0.260 ± 0.01	0.380	0.508	0.326, 0.690	2.3	20.9
RAT_85	0.269 ± 0.02	0.272 ± 0.01	0.274	0.555	0.377, 0.732	4.7	16.3

RAT_86	0.274 ± 0.02	0.277 ± 0.02	0.241	0.561	0.392, 0.730	4.7	11.6
RAT_87	0.281 ± 0.02	0.274 ± 0.01	0.231	0.574	0.403, 0.745	4.7	11.6
RAT_88	0.296 ± 0.02	0.297 ± 0.02	0.433	0.521	0.348, 0.694	14.0	16.3

AUC: area under the receiver operating characteristic curve. C: central. CI: confidence interval. ETDRS: early treatment of diabetic retinopathy study. G: global. GCL: macular ganglion cell layer. I1: inner inferior. I2: outer inferior. IPL: macular inner plexiform layer. MRW: Bruch's membrane opening minimum rim width. N: nasal. N1: inner nasal. N2: outer nasal. NFL: macular retinal nerve fiber layer. NI: nasal inferior. NS: nasal superior. PPAA: posterior pole asymmetry analysis. RAT: retinal average thickness. RETINA: whole retinal layer. RNFL: circumpapillary retinal nerve fiber layer. S1: inner superior. S2: outer superior. T: temporal. T1: inner temporal. T2: outer temporal. TI: temporal inferior. TS: temporal superior.

Table S4. Comparison of Patient Retinal Thickness Using the Optimal Parameters to Distinguish Between Patients With and Without Disease Progression

Scan	Parameter	Thickness (μm) (mean ± SD)		*P*	AUC	95% CI	Sensitivity at 95% specificity (%)	Sensitivity at 80% specificity (%)
		**With disease progression**	**Without disease progression**					
RNFL	T	89.92 ± 39.79	77.62 ± 12.61	0.156	0.558	0.364, 0.751	0.0	12.8
	TI	151.54 ± 29.47	143.38 ± 20.76	0.193	0.567	0.369, 0.765	2.6	5.1
	NI	105.67 ± 15.37	110.71 ± 21.22	0.188	0.418	0.241, 0.594	20.5	30.8
	N	78.17 ± 13.00	78.65 ± 10.59	0.454	0.447	0.243, 0.650	2.6	17.9
	NS	177.67 ± 15.66	123.35 ± 20.52	0.160	0.427	0.243, 0.611	7.7	25.6
	TS	140.71 ± 14.98	133.24 ± 21.15	0.093	0.641	0.476, 0.806	10.3	20.5
	G	101.38 ± 13.51	100.05 ± 8.95	0.377	0.479	0.275, 0.683	0.0	17.9
MRW	T	192.67 ± 34.64	184.50 ± 29.92	0.236	0.472	0.285, 0.659	16.7	16.7
	TI	299.00 ± 58.97	292.78 ± 50.25	0.373	0.460	0.261, 0.660	16.7	25.0
	NI	316.00 ± 48.14	315.8 ± 62.03	0.497	0.513	0.335, 0.690	0.0	8.3
	N	272.13 ± 49.31	280.97 ± 51.74	0.299	0.573	0.389, 0.756	0.0	16.7
	NS	311.92 ± 89.58	310.96 ± 55.99	0.486	0.483	0.264, 0.702	8.3	25.0
	TS	271.63 ± 69.30	257.99 ± 41.73	0.264	0.434	0.218, 0.649	8.3	41.7
	G	264.79 ± 46.13	264.13 ± 37.73	0.455	0.514	0.302, 0.726	25.0	33.3
ETDRS RETINA	T1	319.88 ± 37.40	326.36 ± 36.67	0.302	0.620	0.407, 0.832	8.3	25.0
	T2	276.29 ± 19.61	276.87 ± 21.62	0.499	0.491	0.303, 0.680	0.0	16.7
	I1	323.50 ± 34.42	328.90 ± 23.90	0.310	0.670	0.468, 0.871	8.3	8.3
	I2	279.08 ± 22.52	273.44 ± 16.65	0.217	0.423	0.225, 0.621	16.7	41.7
	N1	330.17 ± 37.97	335.13 ± 30.54	0.342	0.627	0.434, 0.820	8.3	25.0
	N2	307.88 ± 25.95	303.99 ± 18.82	0.319	0.475	0.276, 0.675	16.7	33.3

	S1	328.63 ± 33.89	335.29 ± 34.23	0.280	0.579	0.372, 0.787	8.3	25.0
	S2	291.71 ± 19.72	289.95 ± 19.49	0.395	0.458	0.265, 0.651	8.3	25.0
	C	275.38 ± 65.83	278.09 ± 55.25	0.449	0.572	0.344, 0.799	8.3	41.7
NFL	T1	20.50 ± 9.62	21.22 ± 12.53	0.418	0.540	0.337, 0.742	8.3	16.7
	T2	21.04 ± 4.36	22.26 ± 4.70	0.209	0.662	0.478, 0.846	0.0	16.7
	I1	26.04 ± 13.37	28.37 ± 11.16	0.296	0.713	0.521, 0.904	8.3	16.7
	I2	37.88 ± 11.48	38.35 ± 6.42	0.447	0.563	0.346, 0.780	8.3	33.3
	N1	23.58 ± 11.99	24.35 ± 13.98	0.424	0.544	0.344, 0.743	8.3	16.7
	N2	46.38 ± 13.41	48.81 ± 9.92	0.285	0.639	0.446, 0.832	8.3	16.7
	S1	25.58 ± 9.83	26.82 ± 12.27	0.362	0.593	0.412, 0.774	8.3	16.7
	S2	38.29 ± 9.17	39.60 ± 7.13	0.328	0.598	0.420, 0.777	8.3	8.3
	C	16.16 ± 15.88	14.02 ± 9.83	0.335	0.516	0.359, 0.762	8.3	25.0
GCL	T1	45.29 ± 9.62	44.78 ± 4.65	0.431	0.433	0.210, 0.655	0.0	10.3
	T2	35.04 ± 6.59	33.00 ± 3.89	0.163	0.598	0.369, 0.828	0.0	5.1
	I1	47.38 ± 10.84	48.77 ± 5.73	0.338	0.377	0.157, 0.598	2.6	17.9
	I2	33.04 ± 6.74	30.40 ± 5.51	0.118	0.653	0.433, 0.872	2.6	2.6
	N1	46.13 ± 8.36	47.68 ± 5.97	0.279	0.396	0.198, 0.595	2.6	23.1
	N2	38.29 ± 5.18	37.37 ± 3.63	0.288	0.540	0.320, 0.759	2.6	2.6
	S1	49.08 ± 9.63	49.64 ± 5.74	0.426	0.427	0.226, 0.629	2.6	30.8
	S2	35.13 ± 5.43	34.04 ± 3.65	0.263	0.579	0.341, 0.817	2.6	2.6
	C	19.96 ± 14.32	17.26 ± 10.17	0.276	0.525	0.301, 0.748	2.6	10.3
IPL	T1	40.83 ± 7.51	41.19 ± 6.08	0.441	0.433	0.212, 0.653	2.6	10.3
	T2	32.17 ± 4.75	31.72 ± 3.58	0.383	0.531	0.312, 0.750	5.1	7.7
	I1	38.46 ± 6.92	38.64 ± 3.55	0.466	0.436	0.212, 0.660	2.6	12.8
	I2	27.38 ± 4.75	25.64 ± 4.84	0.142	0.641	0.427, 0.855	2.6	2.6
	N1	40.50 ± 7.80	40.03 ± 3.80	0.421	0.433	0.235, 0.631	2.6	33.3
	N2	30.75 ± 4.28	29.41 ± 2.92	0.164	0.563	0.357, 0.769	0.0	2.6
	S1	38.92 ± 6.93	40.15 ± 5.66	0.291	0.446	0.240, 0.651	2.6	23.1
	S2	28.58 ± 3.82	28.05 ± 3.21	0.334	0.559	0.330, 0.788	2.6	10.3
	C	23.54 ± 12.07	21.65 ± 8.50	0.311	0.541	0.339, 0.742	2.6	15.4

PPAA	RAT_11	0.224 ± 0.02	0.228 ± 0.02	0.235	0.562	0.381, 0.743	8.3	16.7
	RAT_12	0.231 ± 0.02	0.233 ± 0.02	0.330	0.544	0.368, 0.720	8.3	16.7
	RAT_13	0.240 ± 0.02	0.241 ± 0.02	0.422	0.524	0.337, 0.710	8.3	16.7
	RAT_14	0.250 ± 0.02	0.252 ± 0.02	0.345	0.520	0.330, 0.711	8.3	16.7
	RAT_15	0.264 ± 0.02	0.262 ± 0.02	0.375	0.470	0.278, 0.662	8.3	16.7
	RAT_16	0.276 ± 0.03	0.275 ± 0.02	0.472	0.506	0.306, 0.707	16.7	25.0
	RAT_17	0.285 ± 0.02	0.284 ± 0.02	0.458	0.550	0.361, 0.739	8.3	16.7
	RAT_18	0.277 ± 0.02	0.276 ± 0.02	0.457	0.527	0.340, 0.713	8.3	16.7
	RAT_21	0.226 ± 0.01	0.228 ± 0.02	0.319	0.486	0.315, 0.657	33.3	5.1
	RAT_22	0.242 ± 0.01	0.328 ± 0.02	0.267	0.564	0.390, 0.738	17.9	2.6
	RAT_23	0.261 ± 0.02	0.254 ± 0.02	0.136	0.628	0.438, 0.818	5.1	2.6
	RAT_24	0.279 ± 0.02	0.272 ± 0.02	0.163	0.620	0.421, 0.818	2.6	10.3
	RAT_25	0.287 ± 0.03	0.278 ± 0.02	0.154	0.614	0.418, 0.810	2.6	7.7
	RAT_26	0.287 ± 0.03	0.280 ± 0.02	0.205	0.579	0.375, 0.783	2.6	5.1
	RAT_27	0.294 ± 0.03	0.290 ± 0.02	0.359	0.495	0.278, 0.711	0.0	10.3
	RAT_28	0.299 ± 0.02	0.306 ± 0.02	0.202	0.393	0.185, 0.601	2.6	20.5
	RAT_31	0.242 ± 0.02	0.238 ± 0.01	0.242	0.466	0.288, 0.643	8.3	16.7
	RAT_32	0.268 ± 0.03	0.259 ± 0.02	0.177	0.411	0.215, 0.608	8.3	33.3
	RAT_33	0.299 ± 0.03	0.292 ± 0.02	0.227	0.440	0.235, 0.645	8.3	41.7
	RAT_34	0.322 ± 0.03	0.319 ± 0.02	0.406	0.518	0.290, 0.747	8.3	50.00
	RAT_35	0.322 ± 0.03	0.326 ± 0.02	0.338	0.609	0.419, 0.799	8.3	8.3
	RAT_36	0.314 ± 0.02	0.314 ± 0.02	0.465	0.534	0.335, 0.733	8.3	25.0
	RAT_37	0.299 ± 0.02	0.296 ± 0.02	0.331	0.452	0.245, 0.658	8.3	25.0
	RAT_38	0.302 ± 0.02	0.304 ± 0.02	0.387	0.532	0.343, 0.721	8.3	16.7
	RAT_41	0.246 ± 0.02	0.249 ± 0.02	0.320	0.603	0.419, 0.787	8.3	8.3
	RAT_42	0.282 ± 0.03	0.279 ± 0.03	0.391	0.466	0.226, 0.666	8.3	25.0
	RAT_43	0.319 ± 0.04	0.320 ± 0.03	0.469	0.585	0.381, 0.790	8.3	25.0
	RAT_44	0.308 ± 0.05	0.314 ± 0.04	0.368	0.603	0.395, 0.811	8.3	25.0
	RAT_45	0.304 ± 0.05	0.313 ± 0.04	0.284	0.588	0.378, 0.797	8.3	16.7
	RAT_46	0.333 ± 0.02	0.339 ± 0.02	0.258	0.587	0.393, 0.780	8.3	16.7

RAT_47	0.313 ± 0.02	0.313 ± 0.02	0.491	0.522	0.317, 0.728	16.7	25.0
RAT_48	0.289 ± 0.02	0.290 ± 0.02	0.460	0.509	0.321, 0.696	0.0	25.0
RAT_51	0.250 ± 0.02	0.246 ± 0.02	0.282	0.443	0.243, 0.644	8.3	25.0
RAT_52	0.281 ± 0.03	0.278 ± 0.03	0.390	0.466	0.266, 0.666	8.3	25.0
RAT_53	0.317 ± 0.04	0.320 ± 0.04	0.399	0.568	0.367, 0.770	8.3	16.7
RAT_54	0.307 ± 0.05	0.313 ± 0.05	0.341	0.600	0.391, 0.810	8.3	33.3
RAT_55	0.307 ± 0.04	0.315 ± 0.05	0.293	0.568	0.370, 0.767	8.3	16.7
RAT_56	0.337 ± 0.03	0.341 ± 0.03	0.319	0.547	0.348, 0.746	16.7	16.7
RAT_57	0.320 ± 0.03	0.317 ± 0.02	0.369	0.516	0.318, 0.714	16.7	25.0
RAT_58	0.292 ± 0.02	0.292 ± 0.02	0.492	0.459	0.267, 0.653	0.0	25.0
RAT_61	0.245 ± 0.02	0.242 ± 0.02	0.310	0.499	0.286, 0.712	5.1	20.5
RAT_62	0.271 ± 0.02	0.265 ± 0.02	0.213	0.600	0.409, 0.792	2.6	17.9
RAT_63	0.301 ± 0.03	0.298 ± 0.02	0.383	0.544	0.353, 0.734	2.6	17.9
RAT_64	0.324 ± 0.03	0.327 ± 0.03	0.382	0.467	0.265, 0.669	5.1	15.4
RAT_65	0.330 ± 0.02	0.334 ± 0.03	0.283	0.455	0.260, 0.651	5.1	23.1
RAT_66	0.321 ± 0.02	0.321 ± 0.03	0.478	0.510	0.321, 0.698	5.1	23.1
RAT_67	0.309 ± 0.03	0.303 ± 0.02	0.222	0.547	0.356, 0.738	2.6	7.7
RAT_68	0.305 ± 0.02	0.301 ± 0.02	0.253	0.556	0.376, 0.735	5.1	25.6
RAT_71	0.234 ± 0.01	0.234 ± 0.02	0.488	0.502	0.300, 0.704	7.7	17.9
RAT_72	0.252 ± 0.02	0.247 ± 0.02	0.190	0.612	0.415, 0.809	2.6	15.4
RAT_73	0.266 ± 0.02	0.265 ± 0.02	0.398	0.503	0.302, 0.704	2.6	20.5
RAT_74	0.286 ± 0.03	0.286 ± 0.02	0.485	0.447	0.247, 0.647	2.6	23.1
RAT_75	0.298 ± 0.02	0.296 ± 0.02	0.385	0.538	0.339, 0.738	5.1	15.4
RAT_76	0.294 ± 0.02	0.291 ± 0.02	0.336	0.571	0.387, 0.754	5.1	15.4
RAT_77	0.293 ± 0.02	0.288 ± 0.02	0.223	0.595	0.423, 0.767	5.1	20.5
RAT_78	0.300 ± 0.02	0.300 ± 0.02	0.493	0.518	0.342, 0.694	5.1	30.8
RAT_81	0.226 ± 0.01	0.228 ± 0.02	0.364	0.546	0.352, 0.740	7.7	28.2
RAT_82	0.233 ± 0.01	0.234 ± 0.01	0.424	0.416	0.217, 0.614	2.6	33.3
RAT_83	0.247 ± 0.02	0.245 ± 0.01	0.345	0.497	0.286, 0.707	2.6	20.5
RAT_84	0.259 ± 0.02	0.258 ± 0.01	0.434	0.534	0.344, 0.724	2.6	25.6

RAT_85	0.271 ± 0.02	0.269 ± 0.02	0.358	0.590	0.409, 0.771	2.6	25.6
RAT_86	0.275 ± 0.02	0.274 ± 0.02	0.449	0.534	0.356, 0.713	5.1	23.1
RAT_87	0.281 ± 0.01	0.281 ± 0.02	0.459	0.557	0.380, 0.733	10.3	25.6
RAT_88	0.293 ± 0.02	0.296 ± 0.02	0.269	0.470	0.298, 0.642	12.8	35.9

AUC: area under the receiver operating characteristic curve. C: central. CI: confidence interval. ETDRS: early treatment of diabetic retinopathy study. G: global. GCL: macular ganglion cell layer. I1: inner inferior. I2: outer inferior. IPL: macular inner plexiform layer. MRW: Bruch's membrane opening minimum rim width. N: nasal. N1: inner nasal. N2: outer nasal. NFL: macular retinal nerve fiber layer. NI: nasal inferior. NS: nasal superior. PPAA: posterior pole asymmetry analysis. RAT: retinal average thickness. RETINA: whole retinal layer. RNFL: circumpapillary retinal nerve fiber layer. S1: inner superior. S2: outer superior. T: temporal. T1: inner temporal. T2: outer temporal. TI: temporal inferior. TS: temporal superior.
